# Healthcare crisis in Ukraine – worrying consequences of the Russian-Ukrainian war

**DOI:** 10.3325/cmj.2022.63.315

**Published:** 2022-08

**Authors:** Vitalii Poberezhets

**Affiliations:** Department of Propedeutics of Internal Medicine, National Pirogov Memorial Medical University, Vinnytsya, Ukraine

The large-scale Russian military aggression against Ukraine starting on February 24, 2022 has caused the biggest humanitarian crisis in Europe since World War II. Previous stages of the Russian-Ukrainian war, including the annexation of Crimea and war in Donbas, have already affected over 5 million people. Since 2014, in these conflicts 10 000 persons were killed (1), more than 24 000 were injured, and 1.6 million were forced to flee their homes. An extremely high percentage of these displaced people suffer from conflict-related traumatic events (65%), with significantly higher rates among those with Ukrainian ethnic identity (2). However, the total burden of the Russian hybrid war in Ukraine that lasted for almost eight years seems insignificant compared with the number of the people affected during only a few months of the large-scale military aggression in 2022. From February to August 2022, more than 12.65 million people were directly affected by the war; 16.9 million Ukrainians were displaced – 10.3 million refugees have fled the county and 6.6 million have been displaced internally. Hundreds of thousands of people are still trapped and faced with a shortage of food, water, and medicines (3). On August 1, 2022, the United Nations High Commissioner for Human Rights reported about 12 584 civilian casualties, including 5327 killed (4975 adults and 352 children). The real data could be much grimmer because of the delay in reporting from locations such as Mariupol (Donetsk region), Izium (Kharkiv region), and Lysychansk and Sievierodonetsk (Luhansk region), where intense hostilities are taking place (4).

Russian attacks on health care institutions violate the World Medical Association Declaration on the Protection of Health Care Workers in Situation of Violence. According to the World Health Organization data published on August 9, 2022, more than 434 attacks on health care institutions have been recorded, including 366 attacks on health facilities; 72 attacks affected personnel and patients, resulting in death and heavy injuries (5). In addition to attacks on health care, Russian troops have violated the Geneva Convention related to the Treatment of Prisoners of War by capturing Ukrainian military and civilian medical workers. Only in Mariupol, Russians captured about a hundred Ukrainian doctors, who still remain in captivity without being included to released lists of prisoners and without having an opportunity to communicate with their relatives and the Central Prisoners of War Agency (6).

Russian military aggression also affected a number of clinical trials conducted in Ukraine. Many of them are on a hold or have stopped recruiting. The reduced number of clinical trials in Ukraine puts patients in jeopardy because a considerable number of them direly need medicines. For many patients with respiratory diseases, participating in clinical trials was an opportunity to receive high-quality health care that cannot be offered by the National Health Service. As there is no guarantee for the patients’ safety and no trusted ways for data collection, the perspective for new clinical trials opening sites in Ukraine is not realistic, and the future of ongoing trials is unclear, which is a situation that puts thousands of patients in jeopardy (7).

Unfortunately, the devastating long-term consequence of the war on Ukraine’s health care system will result in many deaths among civilians. Notably, Ukrainians are a peaceful nation and Ukraine has never participated in military conflicts before the Russian aggression in 2014. Ukraine gave up nuclear weapons located in its territory according to the Budapest Memorandum on Security Assurances in 1994, being the second country in history to do so. The Memorandum was signed by the Russian Federation and prohibited it from using military force against Ukraine. Nevertheless, Russian Federation violated this Memorandum with the 2014 annexation of Crimea, the war in the Donbas region, and a large-scale military aggression on February 24, 2022. This is why all civilized countries must stand against unprovoked Russian military aggression and do everything in their power to stop the ongoing war in Ukraine.
